# Silent Shadows: Unveiling the Psoas Abscess and Its Treatment

**DOI:** 10.7759/cureus.71993

**Published:** 2024-10-21

**Authors:** Lovingly M Ferrer Ocampo, Jessica Campisi, Chance Haley, Gurpreet Singh, Guillermo M Uy

**Affiliations:** 1 Surgery, Touro College of Osteopathic Medicine, Middletown, USA; 2 General Surgery, Touro College of Osteopathic Medicine, Middletown, USA; 3 Surgery, Garnet Health Medical Center, Middletown, USA; 4 General Surgery, Crystal Run Healthcare, Middletown, USA

**Keywords:** fistulized appendicitis, iliopsoas abscess, psoas abscess, rare complication of appendicitis, recurrent abscess

## Abstract

Iliopsoas abscesses are a rare complication of appendicitis and are associated with high morbidity and mortality without appropriate intervention. Current literature provides sufficient evidence for managing psoas abscesses via antimicrobial therapy, CT-guided percutaneous drainage, and laparoscopic or open drainage as primary approaches. However, there is insufficient data in the current literature for assessing improved patient outcomes with robotically assisted laparoscopic drainage as an approach to treatment. Here, we present the case of a 72-year-old male with a prior history of perforated appendicitis complicated by a pelvic abscess and treated interventional radiology (IR)-guided drain, left partial nephrectomy secondary to renal cell carcinoma, and bilateral hip arthroplasty presenting with signs and symptoms of recurrent appendicitis. CT imaging found a loculated right iliopsoas abscess adjacent to the appendix, which was not amenable to IR percutaneous drainage. Surgical drainage was deemed necessary with a robotically assisted approach, and the patient had improved clinical status after the intervention. Early results show that robotically assisted laparoscopic surgery has been shown to shorten the clinical course for patients via decreased length of stay, faster recovery times, and better incisions cosmetically. Drainage via robotic laparoscopy allows for complete drainage and irrigation, maximizing source control of infection. It is an effective approach for the management of refractory psoas abscesses secondary to appendicitis.

## Introduction

Recurrent psoas abscesses due to appendicitis are rare but challenging conditions to manage. Common causes of this condition are variant locations of the appendix or an abnormal location of a fecalith. Standard treatment typically involves a combination of broad-spectrum antibiotics, surgical intervention such as laparotomy with appendectomy, or CT-guided percutaneous drainage of the abscess [1,2]. We present a case of a psoas abscess that was initially trialed with conservative treatment. Recurrence of this abscess prompted further management and diagnosis and was later found to be related to fistulated appendicitis. Cases involving fistula formation, such as this one, also require takedown of the tract to prevent recurrence. Surgical procedures such as laparotomy, appendectomy, and laparoscopic approaches have been effective in managing more severe cases of recurrent psoas abscesses associated with appendicitis, leading to favorable outcomes and low recurrence rates [3,4].

It has been well established that psoas abscesses can be successfully managed via CT-assisted drainage and laparoscopic drainage [5]. However, the indication for robotic-assisted laparoscopic intervention has not been clear [4]. We present in this study the case of a psoas abscess that failed initial CT-assisted drainage but was successfully drained via a robotic-assisted laparoscopic approach.

## Case presentation

A 72-year-old male presented to the emergency department with right lower quadrant abdominal pain with an extension to his right thigh. He also reported a three-week history of fever, decreased appetite, nausea, and constipation. Three months before the hospital presentation, the patient had perforated appendicitis complicated by a pelvic abscess, which was managed via an interventional radiology (IR)-guided drain. Other pertinent surgical history included left partial nephrectomy for renal cell carcinoma and bilateral hip arthroplasty.

**Figure 1:**
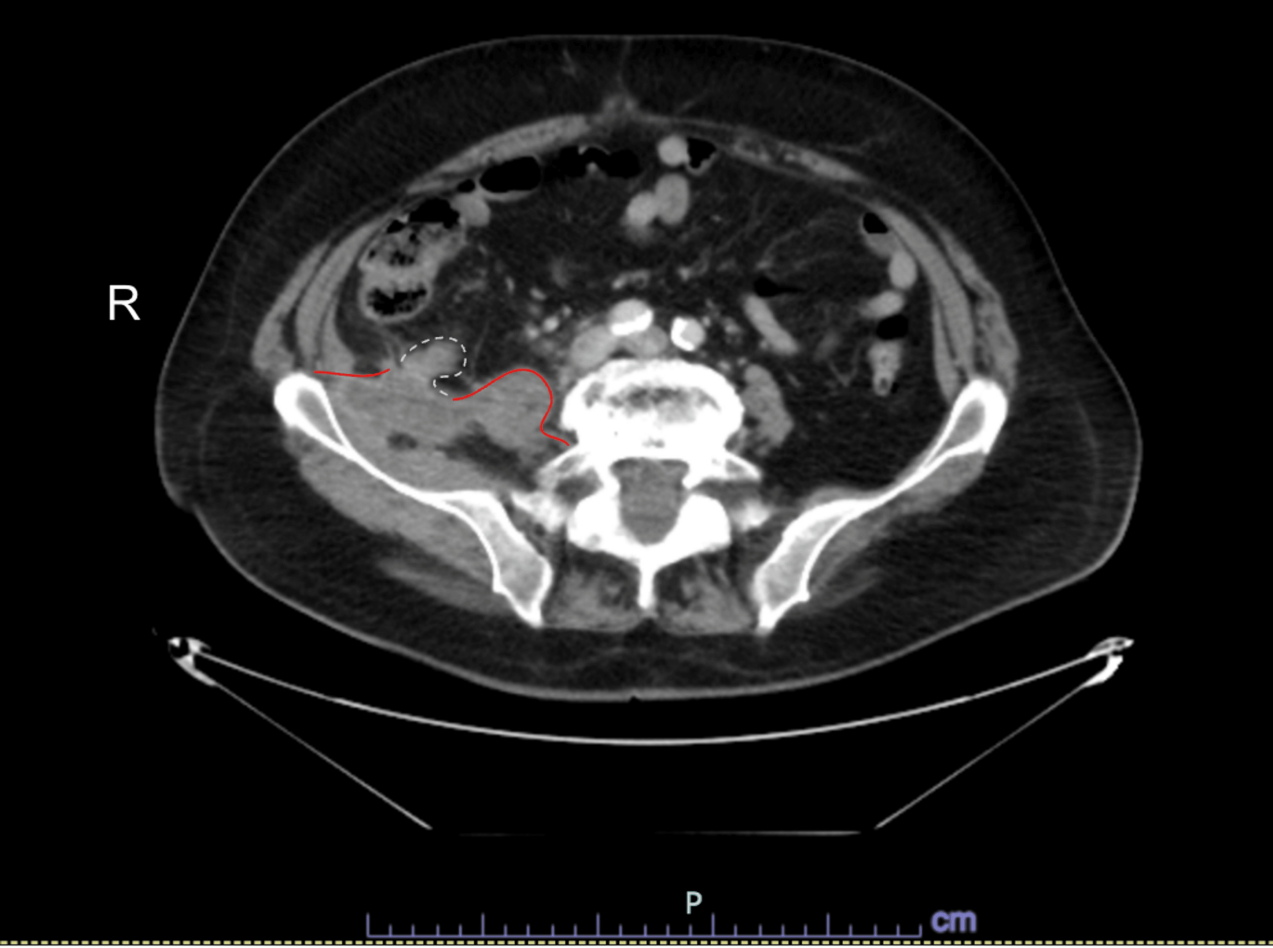
Transverse view CT depicting the appendix fistulized to the iliopsoas accompanied by abscess formation. White dotted line: border of the appendix. Red line: border of the iliopsoas.

**Figure 2:**
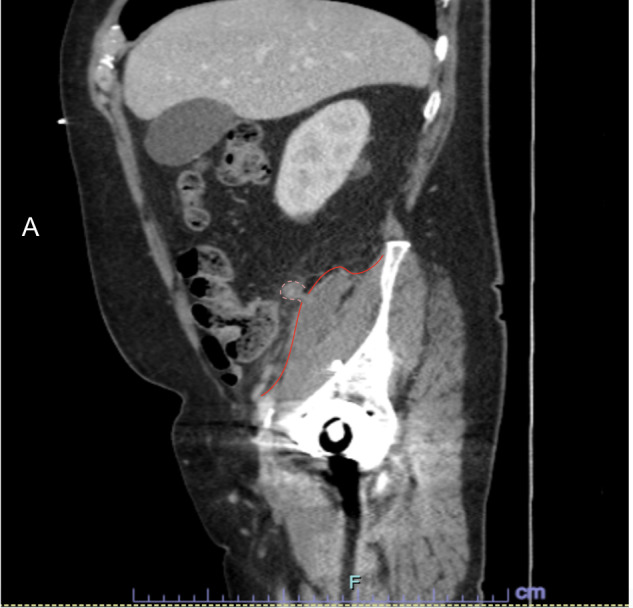
Sagittal view CT depicting the appendix fistulized to the iliopsoas accompanied by abscess formation. White dotted line: border of the appendix. Red line: border of the iliopsoas.

**Figure 3:**
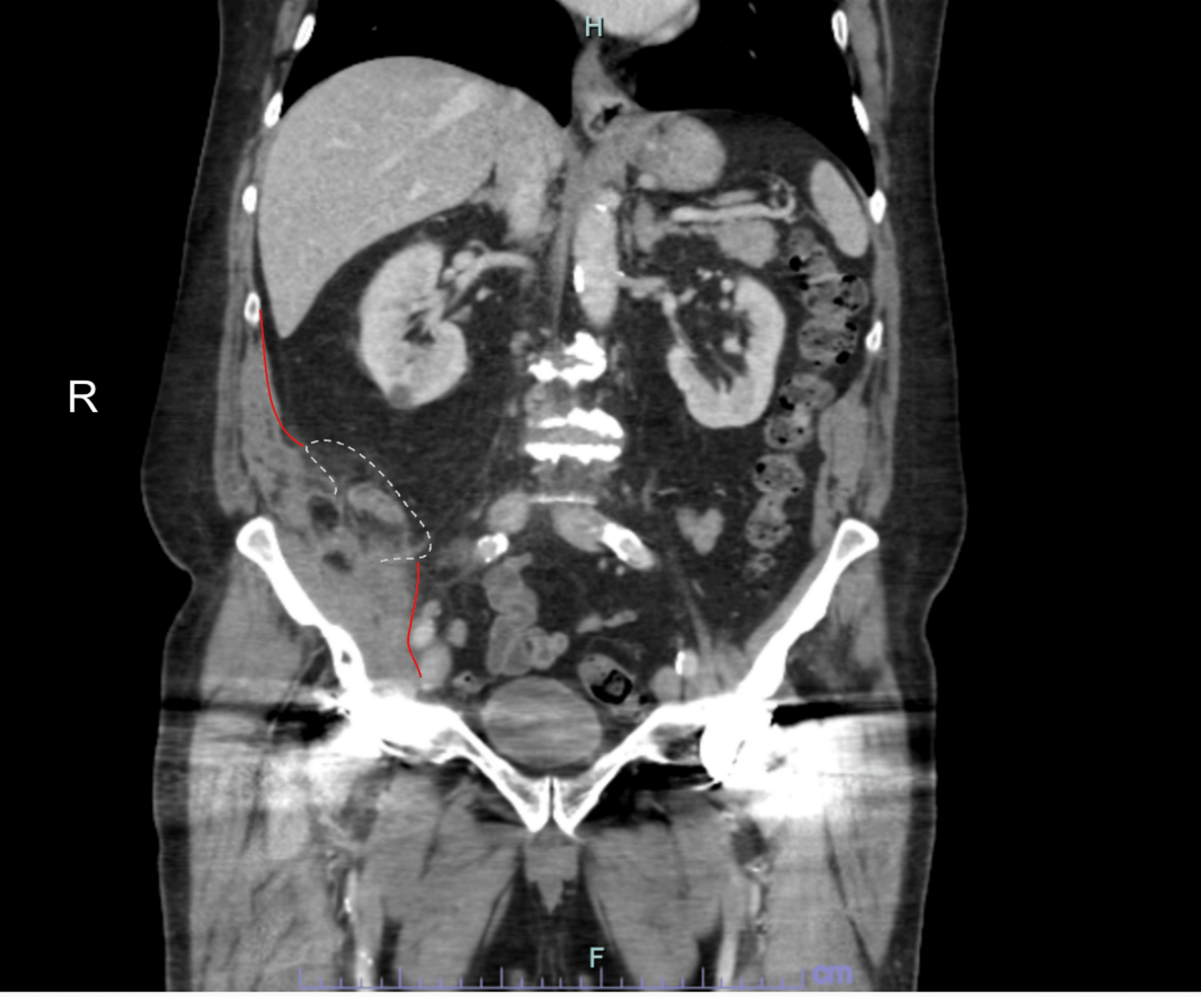
Coronal view CT depicting the appendix fistulized to the iliopsoas accompanied by abscess formation. White dotted line: border of the appendix. Red line: border of the iliopsoas.

On presentation, vital signs were stable, and a physical examination revealed a soft, non-distended abdomen with mild right lower quadrant tenderness on deep palpation, without guarding, rebound, or rigidity. Labs revealed a white blood cell count of 14.3 × 10^9^/L with neutrophilia of 79.9 cells/mm^3^, suggesting infection. A CT scan of the abdomen and pelvis with intravenous (IV) contrast showed a 1.7 × 8.6 cm elongated, complex, and thick-walled fluid collection located within the right iliopsoas, adjacent to the appendix (Figures 1-3). This study, in comparison to a previous scan, confirmed that the size of the abscess had progressed significantly. The patient was admitted for potential IR-guided drainage of the multiloculated abscess but was unfortunately not amenable to imaging-guided drainage. The patient underwent a trial of medical management and was started on IV piperacillin-tazobactam. After three days of conservative management with IV antibiotics, the patient’s condition failed to improve, and he was subsequently brought to the operating room for the surgical management of a symptomatic abscess.

The patient was placed under general anesthesia and positioned in the supine position for an anterior approach using the Da Vinci robotic surgical system to assist in the laparoscopic drainage of the iliopsoas fluid collection with appendectomy. The Veress needle at Palmer’s point was used for insufflation and three additional ports were placed for proficient access to the right iliopsoas area. The appendix was then visualized to be diving into the retroperitoneum. Upon inspection, the appendix was noted to have formed a communication with the large pelvic abscess previously seen on a CT scan. The drainage of the abscess revealed a copious amount of purulent fluid. After suctioning of the purulent fluid, the loculations of the abscess were broken and the abscess cavity was irrigated. Blood cultures preoperatively were positive for gram-positive cocci in clusters; no cultures were taken intraoperatively. The rest of the appendix was then freed from the abscess cavity and procured for pathological study. A 10-French Jackson-Pratt (JP) drain was placed into the abscess cavity in the right lower quadrant and externalized from the left upper quadrant incision. After hemostasis was achieved, the ports were removed and the skin was closed with 4-0 Biosyn sutures and Dermabond. Total operative time was less than one hour and the patient was extubated and transported to the post-anesthesia care unit in a stable condition.

The patient tolerated the procedure well and was hemodynamically stable following the operation. He was placed on a low-fiber diet following the operation. Pathology results were consistent with acute appendicitis. He was continued on intravenous Zosyn and remained afebrile throughout the remainder of his hospital stay. The patient was discharged on postoperative day four in stable condition with a seven-day course of oral amoxicillin-clavulanate and instructions to return to the surgical clinic as an outpatient for JP drain removal. The patient continues to do well and has not experienced a recurrence of iliopsoas abscess.

## Discussion

Psoas abscesses are exceedingly rare with 12 cases reported per year. However, the vague clinical presentation may result in the true incidence of psoas abscesses remaining underreported [6]. Causes of psoas abscesses can be divided into primary or secondary. Primary causes include lymphatic or hematogenous spread of monomicrobial pathogens from a distant source, which classically has been *Staphylococcus aureus*. However, *Escherichia coli* has also been isolated [7]. Secondary causes, which are responsible for 70% of psoas abscesses, are attributed to contiguous spread from an adjacent gastrointestinal, genitourinary, or musculoskeletal infectious source and typically involve enteric bacteria [7,8]. It has been reported that 18% of psoas abscesses present with septic shock, with a hospital mortality rate of 12% [8]. Thus, there is a considerable burden of morbidity and mortality associated with psoas abscess.

With a suggested growing incidence of psoas abscess, and a significant associated mortality rate, we aimed to identify which patients fail initial non-operative management and if novel approaches to psoas abscess management could be successful. Cases of psoas abscess were identified through the use of a literature search using keywords including “psoas abscess” and “surgery.” A retrospective review study examining 48 patients found that 78% of patients were successfully treated with antibiotics alone. However, percutaneous drainage of the psoas abscess was successful in only 40% of patients. Patients who failed management had underlying comorbidities, including an immunosuppressed state, complex diverticular disease, and Crohn’s disease. Of those treated via exploratory surgery, 100% had no recurrence of their psoas abscess [9]. Other studies have found that certain qualities of psoas abscess, such as abscesses with multiple septate or abscesses with locations inaccessible to image-guided percutaneous drainage failed conservative management [10].

We also aimed to determine if a robotic-assisted laparoscopic approach to drainage of a psoas abscess could be considered in surgical management, as discussed in this case study. Currently, no high-quality evidence is available regarding the efficacy of robotic-assisted laparoscopic approaches in the surgical management of psoas abscesses. However, prior studies have shown successful outcomes with laparoscopic drainage. One meta-analysis study found laparoscopic drainage to be more effective in critically ill patients undergoing surgical management of their psoas abscess, as these patients benefited from rapid drainage of their abscess, shorter operative time, and improved recovery times [11]. Given similar indications and outcomes for laparoscopic versus robotic surgery and the success of the robotic-assisted laparoscopic procedure in our case report, we suggest robotic-assisted laparoscopic surgery could be a potential option for the surgical management of psoas abscesses. However, further research is needed regarding this matter.

## Conclusions

Psoas abscesses are a rare complication of recurrent appendicitis. Robotic versus laparoscopic surgical drainage has been shown to lead to similar improvements in patient outcomes in terms of faster recovery times, shorter operative time, and ultimate source control via direct visualization and irrigation. Based on the improved patient outcomes in this report, robotically assisted laparoscopic drainage would be an appropriate addition to the surgical management of refractory psoas abscesses. Further research is needed to fully assess this approach.
